# Supplementary material for: Degenerate conic anchoring and colloidal elastic dipole-hexadecapole transformations
Source: Nat Commun. 2019 Mar 1;10:1000. doi: 10.1038/s41467-019-08645-9 (PMC6397205; doi:10.1038/s41467-019-08645-9)
Supplement: Supplementary file 1 — Supplementary Information [file 41467_2019_8645_MOESM1_ESM.pdf]

Supporting Information

Degenerate conic anchoring and colloidal elastic dipole-hexadecapole transformations

Ye Zhou,[1] Bohdan Senyuk,[2] Rui Zhang,[1] Ivan I Smalyukh,[2,3,4] and Juan J. de Pablo[1,5,*]

[1]*Institute for Molecular Engineering, The University of Chicago, Chicago, Illinois 60637, United States*

[2]*Department of Physics and Soft Materials Research Center, University of Colorado, Boulder, Colorado 80309, United States*

[3]*Department of Electrical, Computer, and Energy Engineering, Materials Science and Engineering Program, University of Colorado, Boulder, Colorado 80309, United States*

[4]*Renewable and Sustainable Energy Institute, National Renewable Energy Laboratory and University of Colorado, Boulder, Colorado 80309, United States*

[5]*Argonne National Laboratory, Argonne, Illinois 60439, United States*[*]

TRANSFORMATION OF SURFACE ENERGY TERMS

In order to gain a better understanding for the **Q**-tensor based surface energy (Equation 1-3), we transform these equations into θ_s-expressions assuming an invariant scalar order parameter S_{eq}. Given that $Q^U_{ij} = S_{\mathrm{eq}}(n_i n_j - \frac{1}{3}\delta_{ij})$, the deviation are provided as below.

$$f^h_{\mathrm{surf}} = \int_{\mathrm{surf}} \frac{W_h}{2} \left(Q_{ij} - Q^0_{ij}\right)^2 \, d\Sigma. \tag{1}$$

$$f^p_{\mathrm{surf}} = \int_{\mathrm{surf}} W_p \left(\tilde{Q}_{ij} - \tilde{Q}^{\perp}_{ij}\right)^2 \, d\Sigma. \tag{2}$$

$$f^c_{\mathrm{surf}} = \int_{\mathrm{surf}} W_c \left(P'_{ik}\tilde{Q}_{kl}P'_{lj} - S_{\mathrm{eq}} cos^2\theta_e P'_{ij}\right)^2 \, d\Sigma. \tag{3}$$

Homeotropic anchoring

In Equation 1 for homeotropic anchoring, the surface preference order parameter tensor Q^{U0}_{ij} with uniform scalar order parameter is defined as $S_{\mathrm{eq}}(\nu_i\nu_j - \frac{1}{3}\delta_{ij})$. Given that $n_i n_i = 1$, $\nu_i\nu_j$ and $n_i\nu_i = \cos\theta_s$, we have

$$\begin{aligned}\left(Q^U_{ij} - Q^{U0}_{ij}\right)^2 &= \left(S_{\mathrm{eq}}(n_i n_j - \frac{1}{3}\delta_{ij}) - S_{\mathrm{eq}}(\nu_i\nu_j - \frac{1}{3}\delta_{ij})\right)^2 \\ &= S^2_{eq}(n_i n_j n_i n_j + \nu_i\nu_j\nu_i\nu_j - 2n_i n_j \nu_i\nu_j) \\ &= 2S^2_{eq}\sin^2\theta_s.\end{aligned}$$

Therefore,

$$\begin{aligned}f^h_{\mathrm{surf}} &= \int_{\mathrm{surf}} \frac{W_h}{2} \left(Q^U_{ij} - Q^{U0}_{ij}\right)^2 \, d\Sigma \\ &= \int_{\mathrm{surf}} W_h S^2_{\mathrm{eq}} \sin^2\theta_s \, d\Sigma.\end{aligned}$$

Degenerate planar anchoring

In equation 2 for degenerate planar anchoring, the term $\tilde{Q}_{ij}^{\perp} = P_{ik}\tilde{Q}_{kl}P_{lj}$ is the projection of $\tilde{Q}_{ij} = Q_{ij} + \frac{1}{3}S_{\text{eq}}\delta_{ij}$, where $P_{ij} = \delta_{ij} - \nu_i\nu_j$ is surface projection tensor.

$$\begin{aligned}\left(\tilde{Q}_{ij}^{U} - \tilde{Q}_{ij}^{U\perp}\right)^2 &= (S_{\text{eq}}n_i n_j - P_{ik}S_{\text{eq}}n_k n_l P_{lj})^2 \\ &= S_{\text{eq}}^2\left(n_i n_j - (\delta_{ik} - \nu_i\nu_k)n_k n_l(\delta_{lj} - \nu_l\nu_j)\right)^2 \\ &= S_{\text{eq}}^2\cos^2\theta_s(2 - \cos^2\theta_s) \\ &= S_{\text{eq}}^2(1 - \sin^4\theta_s).\end{aligned}$$

Therefore,

$$\begin{aligned}f_{\text{surf}}^{p} &= \int_{\text{surf}} W_p\left(\tilde{Q}_{ij}^{U} - \tilde{Q}_{ij}^{U\perp}\right)^2 d\Sigma \\ &= \int_{\text{surf}} W_p S_{\text{eq}}^2(1 - \sin^4\theta_s)\, d\Sigma.\end{aligned}$$

Degenerate conic anchoring

We proposed Equation 3 to represent degenerate conic anchoring, where a new projection tensor $P'_{ij} = \nu_i\nu_j$ is used instead.

$$\begin{aligned}\left(P'_{ik}\tilde{Q}_{kl}P'_{lj} - S_{\text{eq}}cos^2\theta_e P'_{ij}\right)^2 &= (\nu_i\nu_k S_{\text{eq}}n_k n_l\nu_l\nu_j - S_{\text{eq}}\cos^2\theta_e\nu_i\nu_j)^2 \\ &= (\nu_i\nu_j S_{\text{eq}}\cos^2\theta_s - \nu_i\nu_j S_{\text{eq}}\cos^2\theta_e)^2 \\ &= S_{\text{eq}}^2(\cos^2\theta_s - \cos^2\theta_e)^2\nu_i\nu_j\nu_i\nu_j \\ &= S_{\text{eq}}^2(\cos^2\theta_s - \cos^2\theta_e)^2.\end{aligned}$$

Therefore,

$$\begin{aligned}f_{\text{surf}}^{c} &= \int_{\text{surf}} W_c\left(P'_{ik}\tilde{Q}_{kl}P'_{lj} - S_{\text{eq}}cos^2\theta_e P'_{ij}\right)^2 d\Sigma \\ &= \int_{\text{surf}} W_c S_{\text{eq}}^2(\cos^2\theta_s - \cos^2\theta_e)^2\, d\Sigma.\end{aligned}$$

GINZBURG-LANDAU RELAXATION

The minimization of the free energy corresponds to the Euler-Lagrange equation:

$$\frac{\partial f}{\partial Q_{ij}} - \nabla \frac{\partial f}{\partial \nabla Q_{ij}} = 0 \text{ (bulk)}, \tag{4}$$

$$\frac{\partial f}{\partial \nabla Q_{ij}} \cdot \boldsymbol{\nu} = 0 \text{ (surface)}. \tag{5}$$

Applying Equations (4) and (5) to the Landau-de Gennes free energies with a symmetric-traceless operator yields:

$$h_{ij}^{\text{bulk}} = -A\left(1 - \frac{U}{3}\right) Q_{ij} + AU\left[Q_{ik}Q_{kj} - Q_{mn}Q_{nm}\left(Q_{ij} + \frac{\delta_{ij}}{3}\right)\right] + L\frac{\partial^2 Q_{ij}}{\partial x_k \partial x_k}, \tag{6}$$

$$h_{ij}^{\text{surf}} = L\frac{\partial Q_{ij}}{\partial x_k}\nu_k - 2W_c\left(\left(P'_{ik}\tilde{Q}_{kl}P'_{lj} - S_{\text{eq}}cos^2\theta_e P'_{ij}\right) - \frac{\delta_{ij}}{3}\left(\nu_i\tilde{Q}_{ij}\nu_j - S_{\text{eq}}\cos^2\theta_e\right)\right). \tag{7}$$

EXPERIMENTAL MEASUREMENT OF A TILT ANGLE OF CONIC ANCHORING

A director field $\mathbf{n}(\mathbf{r})$ around an elastic hexadecapole is shown in Supplementary Figure 1a. Mismatch between the orientations of an easy axis $\mathbf{n_e}$ at the curved surface of a colloidal particle and a homogeneous far-field director $\mathbf{n_0}$ causes distortions of $\mathbf{n}(\mathbf{r})$, which appear in polarizing textures between crossed polarizers (Supplementary Figure 1b, c) as eight bright lobes around the particle. However, due to combination of the tilted anchoring and a curved particle's surface, there are four regions around the circumference of the particle where a surface normal ν is tilted with respect to $\mathbf{n_0}$ by an angle θ_e and, as a result, a local $\mathbf{n_e}$ at the surface of the particle is parallel to $\mathbf{n_0}$. The director field stays undistorted in these regions $[\mathbf{n}(\mathbf{r}) = \mathbf{n}_0]$ accross the entire sample thickness and they appear in the polarizing micrographs as dark areas separating two bright lobes in the each quadrant of a spherical particle. This allows measuring the tilt angle θ_e of conic anchoring experimentally as shown in Supplementary Figure 1c.

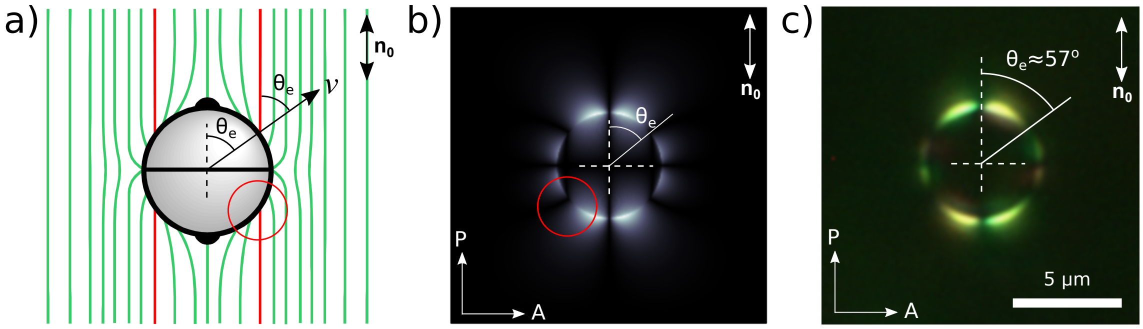

Supplementary Figure 1. Schematic of a director field $\mathbf{n}(\mathbf{r})$ (green lines) around an elastic hexadecapole (a) and corresponding calculated (b) and experimental (c) polarizing micrographs between crossed polarizers A and P. Red straight lines in (a) show the spatial locations where local $\mathbf{n_e}$ is parallel to $\mathbf{n_0}$.

DEFECT CHARGE

The director orientation around the defect (2D), in special cases, may be described by

$$\phi = s\alpha + c \tag{8}$$

where $\alpha = \tan^{-1}(y/x)$, c is a constant, and s is the defect topological charge (Supplementary Figure 2a). As shown in Supplementary Figure 2b, for colloids with rigid homeotropic anchoring, the director orientation on surface follows $\phi = \alpha$, giving that $s = 1$ and $c = 0$.

A similar analyis of both elastic hexadecapole and CA dipole with $\theta_e = 45°$ is performed as shown below below:

- elastic hexadecapole (Supplementary Figure 2c)

$$\begin{cases} s = 1, c = \frac{\pi}{4} & \text{for } \alpha \in (0, \frac{\pi}{2}) \cup (\pi, \frac{3\pi}{2}), \\ s = 1, c = -\frac{\pi}{4} & \text{for } \alpha \in (\frac{\pi}{2}, \pi) \cup (\frac{3\pi}{2}, 2\pi), \end{cases} \tag{9}$$

- elastic CA dipole (Supplementary Figure 2d)

$$\begin{cases} s = 1, c = \frac{\pi}{4} & \text{for } \alpha \in (-\frac{\pi}{2}, \frac{\pi}{2}), \\ s = 1, c = -\frac{\pi}{4} & \text{for } \alpha \in (\frac{\pi}{2}, \frac{3\pi}{2}), \end{cases} \tag{10}$$

Therefore, we may conclude that nematic colloids with degenerate conic anchoring also carry a 2D effective charge of $q = +1$.

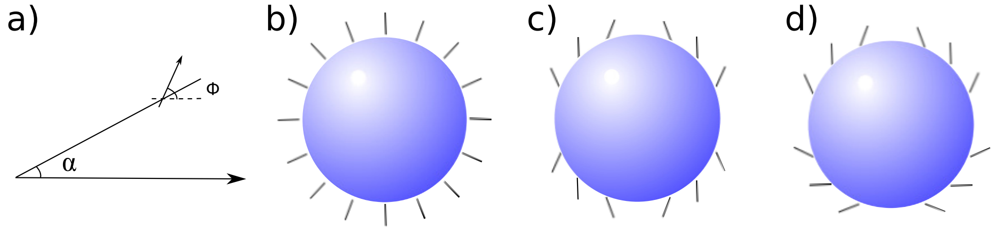

Supplementary Figure 2. a) Director orientation (ϕ) indicated by arrow along a polar line at α. b) Sketch of director configuration on surface for a colloid with rigid homeotropic anchoring. c-d) Sketch for director configurations on surface for colloids with degenerate conic anchoring ($\theta_e = 45°$) for elastic hexadecapole (c) and elastic CA dipole (d).

ANCHORING AND SYMMETRY

It is an interesting question; can a nematic colloid form a hexadecapole with a weak planar or homoetropic anchoring. As shown in Supplementary Figure 2c of the manuscript, the n_x color maps for nematic colloids $\theta_e = 0°$ and $90°$ both exhibit a quadrupole symmetry. A weaker anchoring strength will not change the symmetry, as shown in Fig 4 of Ref. 25. The formation of hexadecapolar symmetry arises from a superposition of the two quadrupoles of opposite sign, and is, therefore, not achievable by lowering the anchoring strength for either planar or homeotropic anchoring.

CHIRAL DIPOLE

In our manuscript, under the one-constant assumption, surface directors of LCs always stay in the meridian plane of colloids. When taking the elastic anisotropy into consideration, the surface director may escape from the meridian plane, where a term which represents an anchoring degenerate in the azimuthal angle - as we proposed - is crucial in order to capture the resulting chirality. Supplementary Figure 3 provides an example of chiral dipole when $k_{33}/k_{22} = 5$ and $\theta_e = 45°$. The angle ϕ is defined as the angle between the director and the meridian plane, which illustrates the twisting power or chirality near defects.

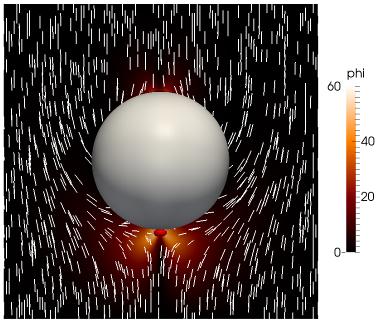

Supplementary Figure 3. a) Director fields of an elastic CA dipole with degenerate conic anchoring ($\theta_e = 45°$) when $k_{33} = k_{11} = 5k_{22}$. The defect is shown in red. The angle phi (ϕ) is defined as the angle between the director and meridian plane.
